# Effectiveness and tolerability of systemic therapies in oral lichen planus: A retrospective cohort study

**DOI:** 10.1016/j.jdin.2024.02.008

**Published:** 2024-02-20

**Authors:** Emma L. Myers, Alison N. Hollis, Donna A. Culton

**Affiliations:** a University of North Carolina School of Medicine, Chapel Hill, North Carolina; b Department of Dermatology, School of Medicine, University of North Carolina at Chapel Hill, Chapel Hill, North Carolina

**Keywords:** azathioprine, drug response, hydroxychloroquine, medical dermatology, methotrexate, mycophenolate mofetil, oral lichen planus, oral mucosa

*To the Editor:* Oral lichen planus (OLP) poses a therapeutic challenge as a complex inflammatory condition.^1^ When topical treatments prove ineffective, systemic immunomodulatory therapies are often employed. However, these medications exhibit variable efficacy and potential adverse effects (AEs).^1^, ^2^, ^3^, ^4^, ^5^ This study aimed to assess the efficacy and tolerability of systemic steroid-sparing therapies in OLP patients that had failed topical treatment.

We conducted a retrospective chart review at UNC Dermatology, approved by the institutional review board, analyzing data from OLP patients seen between January 1, 2011, and May 31, 2022. Patients with a diagnosis of OLP confirmed by chart review were included. Lichen planus without oral involvement or those who trialed multiple systemic medications simultaneously for OLP were excluded. Patients were not excluded if they used concomitant topical medications or short courses of systemic steroids for OLP. Demographic information, medication regimens, treatment responses as indicated in clinic notes, and AEs were collected. Analysis focused on mycophenolate mofetil (MMF), methotrexate (MTX), azathioprine (AZA), and hydroxychloroquine (HCQ) (Table I). Given the small number of patients receiving cyclosporine (*n* = 10), apremilast (*n* = 2) and JAK inhibitors (*n* = 1), analysis of these therapies were not included. Data were captured with RedCap and analyzed using descriptive statistics to assess efficacy and tolerability of systemic medications.

**Table I:** Demographics of patients that trialed systemic medications (MMF, MTX, AZA, HCQ) included in retrospective cohort study

	MMF	MTX	AZA	HCQ
Total number of patients who trialed each medication	18	81	27	37
Demographics				
Age (avg)	63	65	62	67
Male	*n* = 5, 27.7%	*n* = 9, 11.1%	*n* = 6, 22.2%	*n* = 7, 18.9%
Female	*n* = 13, 72.2%	*n* = 72, 88.9%	*n* = 21, 77.8%	*n* = 30, 81.1%
Race				
Caucasian	*n* = 16, 88.9%	*n* = 65, 80.2%	*n* = 20, 74.1%	*n* = 30, 81.1%
African American	*n* = 2, 11.1%	*n* = 12, 14.8%	*n* = 5, 18.5%	*n* = 5, 13.5%
Asian	*n* = 0	*n* = 0	*n* = 1, 3.7%	*n* = 1, 2.7%
American Indian/Native Alaskan	*n* = 0	*n* = 1, 1.2%	*n* = 1, 3.7%	*n* = 0
Native Hawaiian/Pacific Islander	*n* = 0	*n* = 0	*n* = 0	*n* = 0
Not recorded	*n* = 0	*n* = 3, 3.7%	*n* = 0	*n* = 1, 2.7%
Subtype				
Reticular	*n* = 0	*n* = 1, 1.2%	*n* = 1, 3.7%	*n* = 1, 2.7%
Erosive	*n* = 16, 88.9%	*n* = 50, 61.7%	*n* = 17, 62.9%	*n* = 21, 56.7%
Ulcerative	*n* = 0	*n* = 4, 4.9%	*n* = 4, 14.8%	*n* = 3, 8.1%
Not recorded	*n* = 2, 11.1%	*n* = 26, 32.1%	*n* = 5, 18.5%	*n* = 12, 32.4%
Sites of additional involvement				
Cutaneous	*n* = 10, 55.5%	*n* = 49, 60.5%	*n* = 15, 55.5%	*n* = 19, 41.3%
Vulvovaginal	*n* = 11, 61.1%	*n* = 32, 39.5%	*n* = 10, 37%	*n* = 9, 24.3%
Nail	*n* = 1, 5.5%	*n* = 8, 9.9%	*n* = 2, 7.4%	*n* = 3, 8.1%
Scalp	*n* = 2, 11.1%	*n* = 12, 14.8%	*n* = 1, 3.7%	*n* = 6, 16.2%
Ocular	*n* = 1, 5.5%	*n* = 2, 2.5%	*n* = 2, 7.4%	*n* = 1, 2.7%
Esophageal	*n* = 0	*n* = 6, 7.4%	*n* = 2, 7.4%	*n* = 2, 5.4%

*AZA*, Azathioprine; *HCQ*, hydroxychloroquine; *MMF*, mycophenolate mofetil; *MTX*, methotrexate.

A total of 110 patients trialed MMF (*n* = 18), MTX (*n* = 81), AZA (*n* = 27), and HCQ (*n* = 37), with the sum of patients listed under each medication (*n* = 163) being greater than the patient population due to patients who trialed more than 1 systemic immunomodulator. Overall improvement rates, combining partial improvement and complete resolution, were 88.9% for MMF, 70.4% for MTX, 59.2% for AZA, and 64.9% for HCQ; complete remission rates were 11.1% for MMF, 14.8%, for MTX, 14.8% for AZA, and 10.8% for HCQ (Table II). Mean time to reach any improvement ranged from 5.5 to 8.0 months for different therapies, primarily lengthened by dose escalation timelines. AEs were noted in 44.4% for MMF, 46.9% for MTX, 44.4% for AZA, and 21.6% for HCQ, and resulted in medication discontinuation in 27.8%, 16.0%, 22.2%, and 13.5% of patients, respectively. Medication was used as first line systemic therapy in 16.7% for MMF, 80.3% for MTX, 70.4% for AZA, and 85.2% for HCQ (Table II). Overall improvement rates and AEs were not significantly different between treatments.

**Table II:** Rates of overall improvement (combining partial improvement and complete resolution), months to achieve improvement, and adverse effects experienced by patients specifically with oral disease on each medication

	MMF	MTX	AZA	HCQ
Total number of patients who trialed each medication	18	81	27	37
Response to treatment				
Partial improvement	*n* = 14 (77.8%)	*n* = 45 (55.6%)	*n* = 12 (44.4%)	*n* = 20 (54.1%)
Complete remission	*n* = 2 (11.1%)	*n* = 12 (14.8%)	*n* = 4 (14.8%)	*n* = 4 (10.8%)
Overall improvement	88.9%	70.4%	59.2%	64.9%
Months to achieve improvement (Mean, SD, range)	5.9, 2.9, 1.9-12.8	5.5, 3.4, 1.2-14.2	8.0, 4.15, 2.9-13.9	6.2, 2.7, 1.4-10.7
AE∗	*n* = 8 (44.4%)	*n* = 38 (46.9%)	*n* = 12 (44.4%)	*n* = 8 (21.6%)
Number of patients whose AE led to discontinuation of medication	*n* = 5 (27.8%)	*n* = 13 (16.0%)	*n* = 6 (22.2%)	*n* = 5 (13.5%)
Number of times medication was used as first-line immunomodulator	*n* = 3 (16.7%)	*n* = 65 (80.3%)	*n* = 19 (70.4%)	*n* = 23 (85.2%)
Of those who used medication first-line, number of patients who improved	*n* = 2 (out of 3, 66.7%)	*n* = 55 (out of 65, 84.6%)	*n* = 13 (out of 19, 68.4%)	*n* = 16 (out of 23, 69.6%)
If not used first-line, average number immunomodulators trialed and failed beforehand	1.33	1.125	1.25	1.15

*AE*, Adverse effects; *AZA*, azathioprine; *HCQ*, hydroxychloroquine; *MMF*, mycophenolate mofetil; *MTX*, methotrexate.

∗ Most common AEs as follows: MMF: GI disturbances, *n* = 3; MTX: Fatigue, *n* = 18; AZA: GI disturbances, *n* = 4; HCQ: ocular toxicity, *n* = 3 & GI disturances, *n* = 3.

This study shows overall improvement rates of 59.2%-88.9% with commonly used systemic therapies in OLP patients that had failed topical therapy. Unfortunately, complete remission rates are low (10.8%-14.8%), time to response can be long (5.5-8.0 months), and AEs are common (21.6%-46.9%), suggesting that more effective and tolerable treatment options are needed in this patient population. Limitations of this study include the retrospective nature, a relatively small sample size, and that other medications reported to be effective but not used regularly at our site were not thoroughly explored. In conclusion, systemic therapies show efficacy for treatment of OLP, but further investigation of predictors of treatment response, adverse effect rates, treatment durations, and optimal dosing is needed to guide treatment algorithms.

## Conflicts of interest

None disclosed.
